# The Control of Hospital Expenditure with Efficiency

**Published:** 1905-03-11

**Authors:** 


### THE CONTROL OF HOSPITAL EXPENDITURE WITH EFFICIENCY.

In the new Out-Patient Department of Charing Cross Hospital on Friday evening, the 3rd instant, a paper was read by Dr. Donald Mackintosh, Medical Superintendent of Glasgow Western Infirmary, under the auspices of the Hospitals Association. There was a large and representative gathering of hospital chairmen, governors, treasurers, secretaries, and others concerned in hospital administration, the discussion which followed the reading of Dr. Mackintosh's paper being both animated and interesting.

Mr. Thomas Bryant, F.R.C.S., presided.

Dr. Mackintosh, in the course of his paper, said:—

For something like 20 years I have devoted my attention to the study of hospital construction and administration, and during that time I have visited many hospitals in this country and all over the Continent. I believe that if hospital managers encouraged their officials to move about a little more and see what others are doing it would be a very great benefit to their institutions, for a really competent man can go nowhere without learning something. Public interest for some time back has centred very considerably on the difference in expenditure per occupied bed in different hospitals. It is held by some persons that "The comparison per occupied bed is illusory," and I admit at once that it is not an altogether easy matter to discover a just unit of comparison. Nevertheless, the occupied bed calculated on the basis of the total ordinary expenditure has always been regarded as, on the whole, the most practical and fair standard of comparison, and justly so, in my opinion, due allowances being made. Comparisons will indubitably be made so long as hospitals are dependent upon the public for their support and up-keep, and there is no reason why they should not be made.

Taking then the customary and only fair and possible unit of comparison, I find from reference to "Burdett's Hospitals and Charities" for 1905, that the difference in 1903 between the London hospitals and those of the Provinces is very striking. It ranges, in hospitals with medical schools from £50 5s. in Dundee to £142 15s. in St. George's Hospital, London. It is evidently a matter of very great public importance to account for so great a difference, but it is difficult to do so—indeed, it is impossible—unless we are furnished with more details than can be gathered from the published accounts of some institutions. An institution, whether it publish the details of its accounts or not, must be always in a position to furnish them. If, for example, the King's Hospital Fund, which has done so much to assist the London hospitals by administrating money subscribed by the public, were to demand of any hospital why its cost per bed in a particular year is £20, £30, £40, or even £80 more per occupied bed than another, the answer could be readily given if the detailed accounts showed the cost of working each department, and the cost of each item in that department. If extra work had been done in a particular year, or unique difficulties had been met, or some new development had been achieved, or even some new experimental methods tried, the extra expenditure might be held as justified, and the institution would continue to command the confidence of the public. Fortunately, the British public is always ready to pay for efficiency, and it should not be asked to pay for anything else. (Hear, hear.)

### Emphasis on Detail.

I am throwing all the accent upon information as to detail. It is not enough to say, for example, that large sums are spent on certain special departments in this or that hospital, or to enumerate, the *X*-Ray Department, the Bacteriological Department, or the Finsen Light Department, etc., etc., as if these were peculiar to a particular hospital. For inquiry might show that other institutions are carrying on the same good work and utilising the discoveries of the most recent science in the same way and at a much less cost. I would lay down, therefore, as one of the first conditions of effective hospital management that the accounts be kept in such a way that even a layman may be able at any time to see what is spent on each item in each separate department. Now this condition can be fulfilled only if another condition is also satisfied, which, at first sight, seems to be its exact opposite. I mean that the whole work of superintending the various departments should be focussed in the hands of a single administrator, and I need hardly add that he must reside on the spot and be directly responsible for the institution to the board of managers. While each department must have its own competent and energetic head, all the heads must be subordinate and responsible to the general superintendent; otherwise, overlapping and the unnecessary expenditure it entails are not avoidable. A hospital is a single institution although its functions are various. So that the dove-tailing of the departments into one another so far as their administration and expenditure are concerned is a necessity. But, it may be argued, such detail on the one hand and such concentration on the other are impracticable, except in a very small hospital. I should reply, in the first place, that where this condition is incapable of being fulfilled, the hospital is too large. That a hospital must be under one head follows from the fact that it cannot be a collection of independent institutions; and that the superintendent must not be "a merely nominal head of all the departments, or not responsible for the details," is more than obvious. In fact, a head that is "nominal" and ignorant of details, has neither authority nor the right to exercise it. And why should the head be either nominal or ignorant of details in a great hospital? In the case of a large railway company effective organisation has brought it about that the general manager, who is responsible for the whole concern to the board of directors, can lay his hands, when required, upon any detail in the administration; and if this is possible in the case of railways, it should not be impossible in the case of hospitals. Such organisation is as necessary for economy of administration in the one case as it is in the other.

In order to show how far the combination of detailed knowledge with effective superintendence has proved practicable in my experience, I shall take up a few of the main items of expenditure in the Western Infirmary of Glasgow, and, of course, I shall enter into details; for the question at issue is, whether a superintendent can be really familiar with the details of a general hospital.

### Expenditure on Provisions.

First, then, I shall take the question of provisions. It is not enough to be told that a certain sum is being spent on such provisions as butcher meat, fish, butter, and so on. We should be able to say what is the exact expenditure on each of these items in each department; and further, what it costs to feed the patients, nurses, servants, and officials, taken separately. If these details are furnished there can be no difficulty in finding out whether there is waste or extravagance in a particular department, or whether that waste, if it exist, be in the wards or in the nurses' or servants' departments. And I hold that without such accurate and minute details of the actual daily consumption of each item in each separate department, it is impossible to control expenditure in any hospital. The expenditure under this heading of provisions, must, of course, vary to some extent from year to year; but it is both a sign and a result of minute knowledge and supervision of details that the variation should be a minimum. I find, for example, that in the Western Infirmary of Glasgow, the daily average cost for food has only varied ⅜d. per patient per day during the last 12 years; that the average cost for food per day for nurses and servants has only varied 2d. per head per day during the same period; and lastly, that taking the average for food for the whole establishment, it has varied 1½d. per head per day. The speaker then went seriatim through the figures showing the comparative quantities used and prices paid in 1902–1903 and 1903–1904 for butter, bread, ice, tea, meat, milk, wines and malt liquors, and then continued: I have dealt with this item of provisions because the supervision of it requires no professional knowledge, it is one of the simplest items, and best lends itself to this test of figures. But I will show later that every department upon which the necessary knowledge and constant attention are brought to bear lends itself to the same treatment; in short, as superintendent of the hospital I consider it my duty to be in a position to know such details regarding expenditure on every item in each separate department and to have each department so organised that by reference to the books I can supply such detailed information at any time. Indeed, I am at a loss to know how an institution can be properly administered and the expenditure controlled unless some such system for the central management of details is adopted. Before passing from this question of provisions I should like to refer to a paragraph which appeared in the *Standard* of January 19th. The following are the words, and they are attributed to the Hon. Sydney Holland:—"Some of the Scotch hospitals are managed very cheaply and we want to find out how they do it. Of course we cannot pledge ourselves to the adoption of all Scotch methods, for instance, we should not think of feeding our people on porridge. . . . We cannot starve our patients."

Gentlemen, I would readily admit that some Scotch hospitals are managed very cheaply, but I am not in a position to say that the patients are badly fed or starved either in Scotland or elsewhere. I should require detailed and incontrovertible information before I could credit such a charge. Can Mr. Holland supply it? I admit that the diet table of the Western Infirmary shows that the patients on ordinary diet get porridge and milk to breakfast, and if they wish it to supper also, but I cannot admit that porridge is an unwholesome food or unpalatable to a Scotchman. (Hear, hear.) Besides, porridge does not constitute the whole meal in the hospital any more than in the best London hotels; the patients have bread and butter, with tea or coffee in addition, and as much as they desire, and very frequently they have an egg. For dinner they have soup, and in addition either boiled beef or mutton, with potatoes and bread, quite apart from such articles of food as are ordered by the doctor, for instance, chop, chicken, custard, and extra milk, and so on. I do not wish to make any invidious comparisons, but I can answer for the Western Infirmary, where I am the administrator, that we supply our patients with everything they require during their residence, including tea, sugar, and butter; that we do not feed them on foreign meat; that we only use milk which comes in sealed cans direct from a selected and regularly inspected farm.

Dr. Mackintosh then proceeded to fully discuss the following questions:—

(1) Should Galenicals be Bought or Made?
(2) The Supervision of Surgical Dressings.
(3) Medical *versus* Lay Superintendent.
(4) Hardware, Crockery, Brushes, etc.
(5) Heating and Lighting.
(6) Co-operation with Staff and Board.

Now, in the first place, I maintain that no hospital should be satisfied with merely weekly returns. In the wards of each of the surgeons we have in the Western Infirmary adopted a system of books, which is not only an effectual check on waste, but a valuable and easy means of comparison of the amount of dressings used from day to day, week to week, or month to month. Every morning the sister in charge of a set of wards, say under Surgeon X, sends down his books to my office with the details of his requirements for the following day, which I initial. But if there is any exceptional demand I reserve my decision until I have been satisfied that the surgeon himself has really asked for the excess which is indicated in the requisition book; and the columns are so arranged that I can do this at a glance, I can not only see what is required for the day but compare it with any other day that month or previous months. At the end of the month these figures are all converted into money, so that every member of the staff from the senior to the junior can get an accurate idea of what every item has cost under the heading of Surgical Dressings. Returns furnished at the end of the week will only let you know what has already been spent, but in order to show whether there has been use or abuse you require this daily knowledge of each detail. The last column in the schedule of each ward contains the total consumption for the month of each item, and is converted into cash, so that every member of the staff can know precisely on any day and for any month as a whole what is being done.

It has been stated that "one surgeon will use dressings costing double as much as another for exactly the same class of work," and it is argued that this could not be prevented without interference on the part of the superintendent, which men of the highest eminence would not brook. I have to remark, in the first place, that before making any such statement, there should be evidence produced of its truth. As a fact I am prepared to prove its contrary so far as regards the Western Infirmary of Glasgow.

But you will say, that in order to be able to furnish such knowledge of each item of expenditure in each ward for each day and for all the year, an army of clerks would be necessary in a great hospital. We have on an average 440 occupied beds in the Western Infirmary, and each department keeps its books up to date, and my clerical staff consists of two persons, a clerk and a typist.

I doubt very much whether the detection of unusual expenditure under this heading is possible to a lay superintendent. I should maintain on the contrary that it is only a man who has himself been a resident medical officer, and knows by experience what surgical dressing implies, who can suggest economies that are practical and do not sacrifice efficiency to the junior medical and nursing staff who have the immediate charge of the dressings. It must not be taken for granted that a medically-trained man is necessarily devoid of administrative capacity, nor must it be assumed that, in order to effect economy he will interfere with the treatment of the surgical patients in the wards. Every one with any knowledge of hospital administration is aware that it is really the junior surgical officers and nursing staff to whom the credit of economy or the discredit of extravagance is primarily due; so that it is usually sufficient, in the first place, to direct their attention to any unusual expenditure and to put them in a position to know the comparative cost of the material and the comparative expense of the different wards, and if this course does not lead to a change, which it generally does, the attention of the surgeon himself can then be directed to the matter. Otherwise the surgeon might be accredited with extravagance of which he had no knowledge and for which he was not responsible. Nor does this imply the least interference with the methods of a surgeon. It should be unnecessary to say that any demand whatsoever for dressings that comes direct from the surgeon-in-charge is immediately responded to, and done so without comment or criticism. I believe, gentlemen, that a layman in charge would be obliged either to interfere ignorantly or not to interfere at all. In the first place he has not the practical knowledge as I have already indicated, and in the second place it is not probable that he would be listened to by the junior members of the staff with the same respect as a qualified member of their own profession. Here, as elsewhere, if the authority is to be real it must be well informed.

And now we come to the last item of expenditure to which I shall refer, namely, heating and lighting. A large sum is spent in every hospital under this heading, and in forming a comparative estimate it is necessary, of course, to take into consideration not only the relative size of the hospitals, but the different cost of coal, which in London is considerably greater than in the provinces. But the comparative cost of coal in different places is by no means the only matter that requires attention; the quality of the coal that is used for each of a number of different hospitals, and further, the manner in which it is used, are subjects which require close and intelligent supervision. I am persuaded that in these respects it is possible to effect important economies without the sacrifice of efficiency. Let me illustrate once more. In the Western Infirmary we have large boilers for generating steam, and this steam is utilised for heating, cooking, working lifts, working a laundry which turns out every week over 20,000 articles, and by means of the same boiler power we generate all our own electricity, with which we light not only the building, but the grounds, and, in addition, keep going a number of electric ventilating fans and electric lamps for the treatment of lupus. A record is kept of the work done by each engine in every hour of the day and night; and also of the boiler pressure, the steam pressure, the temperature of the engine-room, the amount of fuel consumed and the amount of ashes removed. We generate our own current at a lower cost than if we took it from the public mains. As a matter of fact it costs us 1d. per unit for everything, while if we were to take it from the Corporation mains it would cost us $3\frac{1}{2}$d. for lighting and 1d. for motive power. If we allowed 10 per cent. for depreciation, then our cost would be $1\frac{1}{4}$d.

This method has other great advantages. The current comes to the building at a lower voltage. It can be used directly for medical purposes at any bed in any ward. It is under complete control, and can always be relied upon. How then, you will ask, do we get our heating, and lighting, and cooking, and motive power, and other electrical uses from the same boilers? It is by taking full advantage of fuel economisers. We calculate that by means of these we save an extra boiler, and this, of course, carries with it not merely a lower consumption of coal, but less labour and expense in firing boilers, in cleaning them, and in removing the ashes. I observe that one gentleman mentioned in the recent correspondence in the daily press that in some of the London hospitals you were obliged to employ "an army of engineers, stokers, porters, *et hoc genus omne*." We employ two engineers, three stokers, and a relief man. I am not maintaining that every hospital should generate its own electric current; the site may be too limited to accommodate a power station. Under such circumstances it may very well be better for the managers to take their current from the public mains, and there should be no difficulty in the case of a large hospital in obtaining special terms. I understand, for instance, that in the Edinburgh Royal Infirmary one penny per unit is paid for motive electric power and 2¾d. for lighting.

I would add and accentuate the fact that no administrative official, however competent, can accomplish this work, if, in addition to energy, he has not also the good sense to know how he can secure efficient heads in the various departments, and under what circumstances and within what limits he can leave them a free hand. Good organisation implies willing and loyal co-operation. But further, the superintendent must have the confidence and support of a properly constituted, business-like and representative board of managers. I believe that in this respect all the Scotch hospitals are most fortunate. The board of the Western Infirmary consists of 27 directors, and meets regularly every week. Three of this number are appointed by the Town Council, four by the University, two by the Trades' House, two by the Merchants' House, two by the Faculty of Physicians, two by the legal profession, two from outlying burghs and 10 from the general contributors, of whom two are ladies and two are working men. You will agree with me that such a board represents all the various interests that are centred around a large hospital. Their management, from an administrative point of view, is in every sense most satisfactory. Many of the managers are connected with the largest business concerns in the City, and they give without stint to the hospital the advantage of their varied knowledge and experience. For years, to take one instance, in making the purchase of linen I have had the assistance of more than one manufacturer of linen, and they have not only devoted their own time and attention to the purchase of the goods, but placed at our disposal their staff of experts. The result is that at all times we can buy in the best market and at the best prices.

Sir Henry Burdett in an article in *The Times*, of February 1st, speaks as follows:—"Within the last few days I have been approached by some of the managers of the largest and most important enterprises, responsible for the purchase in the best markets and for the supply of goods of all kinds to the public, who are prepared to place the machinery and organisation of their large businesses and the services of their staff at the disposal of the hospitals in an absolutely honorary capacity." It is not for me to ask whether this offer has been accepted or not by any of the London hospitals, but I may say that the Western Infirmary of Glasgow, by the very constitution of its board and their loyalty to the institution, has, throughout my experience, enjoyed this great advantage. The economy of expenditure that is thus secured is too obvious to require that I should dwell on it. It is possible that certain advantages might follow from the combination of the smaller hospitals in the purchase of goods, but it seems to me clear that as regards the large hospitals the most economical and effective method by far is that of making use of the information and guidance of experts who are members of their boards. And, most certainly, every large hospital should at all times be free to purchase its goods in the open market.

### Economy in Use.

But, after all, economy in purchase is only one aspect of the problem. Economy in use is probably of even greater importance. And this can be secured only by means of that constant supervision which I have tried to bring before you. Such supervision, it seems to me, cannot be exercised by the board of managers either individually or collectively; neither can it be done by the secretary, who of course has to attend meetings of managers and committees, keep minutes and collect the necessary funds for the upkeep of the institution. The secretarial and administrative work are through and through distinct; the administrator must be present at the hospital, but in most Scotch hospitals the secretary has his office in the centre of the city, in the middle of the business quarters, where he is in constant touch with business men in every department of commerce and manufacture. (Applause.)

### THE DISCUSSION ON EXPENDITURE.

The Chairman said they had listened very attentively to a remarkably interesting paper which showed what might be done by a hospital superintendent who was not above learning all the details of his work. He invited discussion, in the course of which no doubt many of the lessons to be learned would be emphasised and elucidated.

Mr. Conrad Thies, of the Royal Free Hospital, said the first point on which he would like to ask for the opinion of the lecturer was as to the size of the hospital for which he recommended the employment of a superintendent, medical or lay, apart from the secretary? He had advocated that the secretary's department should be separate from that of the administrator of the internal affairs of the hospital, and no doubt for a hospital of any size such an arrangement would have advantages. But very many of the smaller institutions could not afford to pay the salary of a competent superintendent and of a secretary as well. It must be borne in mind that they had in London a notable example of a successful medical superintendent, but they had many successful lay managers also. It seemed a surprising thing that a successful medical man should be content not to follow his own profession but to take up with hospital management. It seemed to him a counsel of perfection what Sir Henry Burdett had recently recommended, that it was desirable that hospitals should be managed by men personally acquainted with the details of the cost of the various requirements of the different departments. Personally, he thought that with a small hospital of from 200 to 250 beds it would be impracticable to have a superintendent responsible for the internal administration and also a secretary responsible only for secretarial duties, and that it was quite possible for the secretary of a hospital of moderate size to be acquainted with the most minute details of the whole organisation. Indeed, he thought there were distinct advantages in having the administration and secretary's department combined. It was quite possible, if they had a board who would attend, to lay before them statistics week by week showing the cost not only of every patient, but of every nurse and every officer. By that means alone could an adequate check be kept on expenses. It was quite useless to find out at the end of a quarter that something was not as it should be. That was the system they had at the Royal Free, and he thought probably at most of the smaller hospitals. The Board of Management should be acquainted with the cost of every article in use—alcohol, drugs, dressings, etc., and to that end the medical officer attended the weekly meetings, and the steward also was questioned with regard to coals, provisions, and so forth. Dealing with comparisons of cost as between London and the great Northern hospitals, there were many considerations to be taken into account. First, as regards heating, lighting, and power. He was looking the other day into the accounts of his own hospital, and found that the expenditure on warming, lighting, and power in the laundry was one-seventh of the whole cost of upkeep. They had to pay a high price for coal—two-thirds more, or double what they paid in Glasgow; they were obliged to have coal without smoke; they had to pay for their current, the hospital not being large enough to make electricity for themselves economically. Then, outside London hospitals were treated very lightly by the local authorities. In London, especially in the case of St. Thomas's and other of the more recently built hospitals, they paid enormous sums to the local authorities. Then as to water: at the Royal Free, under an old arrangement, they used to pay £40 a year; now they paid £200, and it was quite possible that under the new Water Board they might find themselves worse off instead of better. All these things made a great difference. Dealing with another point raised by Dr. Mackintosh, he noticed that he referred to the important consideration that some hospitals did not provide everything required by their patients. One of the most progressive London hospitals, to which they looked for light and leading, refused to give their patients tea, sugar, or butter, and on the basis of the Royal Free's expenditure this would mean a difference of £1,400 a year in outlay. In days gone by patients were supplied with beer *ad lib.*, and to-day some hospitals supplied beer and wines when ordered —champagne and so on—and yet did not supply essential items like tea, sugar, and butter. It was an anomaly maintained by custom. These were a few of the points that occurred to him out of a paper full of interest and instruction.

The Hon. Sydney Holland said he wished to thank Dr. Mackintosh for the paper he had read, and would like to clear up one misunderstanding with regard to himself. He did not wish to imply that the Scotch hospitals starved their patients. What he meant was that if they asked Englishmen to eat porridge they would starve on it. If they were offered it at the London the patients said, "No, thanks; we can get skilly in prison for nothing." (Laughter.) He had actually offered a reward if they would eat porridge, but they absolutely refused. Coming to the principles of the paper, he entirely agreed with the proposition that, while each department had its own head, they must all be under one chief administrator. He thought they were all agreed about that. But when the lecturer asked why should the head of all the departments be ignorant of details, he would point out that there was a great difference between being "ignorant of" and "not responsible for." Of course, the head must be responsible for every detail, but, taking the lecturer's illustration of railway management—and, since he (Mr. Holland) was deputy-chairman of a railway and docks company, he could speak with knowledge—the general manager of a railway could not be responsible for every detail. He could not be expected, for instance, to answer a question offhand about the grease used for the axles; if such a question arose he would send for the man who was responsible for the grease and get the answer from him. So in a great hospital like the London, with close on 900 beds the head must look to subordinates for precise details; but he entirely agreed with the system the lecturer had set forth, and as a fact all such statistics as he had enumerated were known in their secretary's office just as in Dr. Mackintosh's hospital of half the size. The difference was in the name by which they called their head, and it really made no difference to his efficiency whether he was called a secretary, a house governor, or a superintendent. The paper they had heard brought forward two important considerations; one that in every hospital there should be a "Boss," the other the question whether he should be a medical man or a layman. With the first point he entirely agreed; on the second he ventured to differ very strongly from the views he appeared to advocate. He (Mr. Holland) did not lay down the proposition that a medical man must be devoid of administrative capacity; what he objected to was the claim put forward, as in the statement made by Sir Henry Burdett that if a medical superintendent were put over the London Hospital they could reduce the cost per bed by £20.

Sir Henry Burdett: £30! If you will let me put in a medical superintendent I will prove it.

Mr. Holland said Sir Henry knew that was impossible. It was like others of his statements—vague, unnecessary, and inaccurate! What he (Mr. Holland) maintained was that what was wanted was a good administrator, in whatever profession he might have been brought up. Why shouldn't an engineer be a good hospital superintendent, or any one else who had the faculty for organisation? There was really nothing in the fact that a man had taken up with a career and had not gone on with it. If a medical man found he had made a mistake, and, not being a success as a doctor, was content with £300 or £400 a year, was that man of necessity the right man to manage a hospital? He would rather go to a man who had been successful in his own walk of life. Dr. Mackintosh had said that a layman couldn't know if there were waste of bandages; yet he admitted that he could have nothing to say to his surgeons, and that it was only among the house men and the poor nurses that he could prevent waste. Take a case in point in London. At Guy's they had as superintendent Sir Cooper Perry, a medical man, and an exceptionally able man, yet his cost for bandages was no lower than the other hospitals—indeed Bart's—poor much-abused Bart's—was lower than Guy's. It was a thing anybody could check. Did a superintendent's being a medical man teach him to buy food well? was it good for laundry or electricity or engineering or carpentry, or any of the other matters he had to supervise? Had he not shut himself up for five years of his life in the study of the human body? He emphatically disagreed with the idea that a hospital superintendant must be a medical man. If Sir Henry Burdett dared to show his face in print and say that Guy's was better managed than the other London hospitals, he had figures which would knock him into a cocked hat. In provisions, in alcohol, in surgery, and in domestic service they were behind the rest, while their salaries and wages were higher than King's and the Royal Free. The medical superintendent of Guy's was "one of the best," but even he had not succeeded in making it a pattern to the rest for economy. As to Dr. Mackintosh himself he thought his success as an administrator at Glasgow had been not because he was a medical man, but in spite of it. It was not the medical superintendent that made the Scotch hospitals so cheap. At the London they had just been doing as the lecturer recommended, and sending some of their officials round to visit them, so he could tell them of some of the advantages they enjoyed. Dr. Mackintosh could get home-grown Scotch meat at 3¾d. per lb; at the London they had to pay 4¾d. or 5d. In Glasgow they got their window-cleaning done for £26; at the London it cost them £300. The difference might be due to the clearer atmosphere, or to the fact that they didn't mind dirty windows. Then in Glasgow they got their water free; at the London their water rate was over £500. They paid 12s. 6d. for coal, as compared with the London 16s. 10d., and for their furnaces 8s. 6d. a ton as against 20s. Their rates and taxes were £35, compared with £820, and while the Glasgow Infirmary got its telephones free, at the London they paid £325. Their stokers worked a 12-hour shift; "ours won't," said Mr. Holland. Then in Scotland they gave no pensions; at the London they paid £2,200 a year. That did not seem to him unreasonable. There were other ways in which the two institutions differed. Dr. Mackintosh could get his staff to pay their visits in the morning so that he had the afternoon free for cleaning, etc. When the deputation visited him, he was able to take them round and show them over the place quite peacefully, but he could not imagine a London secretary going about with people without being wanted every few minutes. In fact their whole atmosphere of rush and activity was so different that in comparison a Scotch hospital always struck him as a haven of rest. Dr. Mackintosh did no massage; at the London they had 13,000 cases in the out-patient department alone; and he had no Eye or Dental departments. He was not putting these differences forward to depreciate the Glasgow Infirmary but to show that there was no fair comparison. One other point he would take. Dr. Mackintosh thought it was a mistake to make their own drugs and that the practice was dying out, but, on the contrary, it was a new system which was being taken up and the time was coming when all large hospitals would do it. Their experience was that it paid them over and over to make their own drugs.

Sir Henry Burdett said the key to the most excellent paper to which they had listened that evening was undoubtedly the statement that

> "After all, economy in purchase is only one aspect of the problem. Economy in use is probably of even greater importance, and this can be secured only by means of constant supervision."

In his opinion, the whole question of control must depend on accurate technical knowledge, and he would remind them that he himself had, as a layman, been the superintendent of different large hospitals for many consecutive years, and he had not, therefore, become converted to the belief that it was essential for economy and efficiency that the internal arrangements of all large hospitals should be under the control of a medical superintendent without grave consideration and full knowledge. But at that time it was possible to a great extent for a layman to exercise efficient control because there had not then been the remarkable developments in treatment and in results which in recent years had not only greatly reduced mortality, but practically increased the number of beds by enabling a great many more patients to be treated since the length of stay had been materially curtailed. In consequence, hospital control had in many ways become much more technical and important. Moreover, they could not in voluntary hospitals divorce the question of finance from that of management. With the great development in methods of treatment that had been going on there had come increase of expenditure, and that meant an enormous extra strain on the secretary and his staff in the effort to raise necessary funds to afford the hospitals adequate support. Hence there were now required two officers in place of one—*i.e.* a medical superintendent and a secretary—at every large hospital. In these days, when a hospital had to raise 10 or 20 or even 40 or 50 thousand pounds a year in voluntary gifts from the public, and it had a secretary able to accomplish that, it was quite clear that he had, with the ordinary work attaching to the secretarial department, quite enough to do, and that he ought to be properly and adequately paid for it. His (Sir Henry's) feeling was that in London the cost per bed was so much higher than in many other places because too little was spent on salaries. If they wanted to bring about a reduction of £30 in the cost per bed they must do more in that direction. It had been abundantly proved in the best managed businesses that there was no item of expenditure on which it was wiser to be lavish than the remuneration of the men whose brains were capable of making it a success. His idea was that there should be a competent medical superintendent in control of the internal arrangements of the greater voluntary hospitals, because the lay secretary had more than enough to attend to in his own immediate department. Mr. Holland had asserted that a medical superintendent had no more experience than a layman; but surely during the last three years of his medical course his training and hospital experience in the wards and all departments of treatment must fit him to exercise effective supervision over those engaged in the internal administration. It cannot be said that the average administrative ability of medical men is any less than that of men in other ranks of life. On the contrary, there are to be found as able or abler administrators amongst the medical superintendents of the asylums all over the country as in any other class of men in charge of large businesses or establishments anywhere. A medical superintendent is a man who professionally knows much of what is essential in the work he has to supervise, and should never be "a nominal head, ignorant of details," although a layman must at first necessarily be so. It is impossible, too, for a layman to take effective charge of a hospital if he is to be responsible for its finances, and at the same time to educate himself in technical details, so as to enable him ultimately to qualify, while he has all along to act as superintendent. He had heard it said that it was now easy to control a great institution because of the facilities afforded by the telephone, and that all that had to be done was to switch on each department to the central office, and that so the secretary could look after everything. (Mr. Sydney Holland expressed dissent.) But the whole teaching of the paper to which they had listened with so much interest was that to get efficiency and economy they must have a superintendent who would, day by day, go through the whole institution and keep his eye on everything. The superintendent must have every item dissected in his books, so that he could check it and compare it with the previous week or month or year—could ascertain on the spot the cause of any difference shown and decide whether it was justifiable or not. He (Sir Henry) had understood Mr. Holland to say that they had at the London Hospital the same system in vogue as that described by Dr. Mackintosh, but he believed that the great difference was that the London had weekly or monthly records and comparisons, not daily. No one who had not followed these matters closely, as he had, could realise what a difference in system this represented, because with daily records everyone had brought home to them the consciousness of continuous control. A medical superintendent, free from all the worry connected with the support and maintenance of a voluntary hospital dependent on the public for funds, could each day bring to bear on every department, and upon the whole internal arrangements of the hospital, that continuous control and expert oversight which was essential to economical working. Control so exercised was beneficial alike to those who did the work and to the patients for whom it was done. Such a system as Dr. Mackintosh had outlined to them made for efficiency and the comfort of all concerned, as well as for economy, particularly in regard to the admission of patients at times when necessarily none but juniors were available. They were all much indebted to Dr. Mackintosh for the very lucid, detailed, and effective way in which he had shown how it was possible to exercise control, and how the principles of economy could be applied to the problem of the reduction of the cost per bed. They would all agree that what had been said was worthy of the closest attention. They in London had been so much concerned in the raising of money, and so gratified and cheered by the results of Professor Lister's system on the patients, that they had to some extent overlooked the question of cost. Now, however, that question of cost required their most earnest attention and closest inquiry. After all, it did not matter whether they considered this system or that the best; what was wanted was some common basis of comparison between each hospital and every other one similarly circumstanced and working under like conditions. The able paper that had been read, and the discussion that had followed, must direct attention to these important and pressing questions, and could not fail to be very instructive to everyone who had the best interest of the voluntary hospitals at heart.

Mr. William Morton (Mount Vernon Hospital) said they had been greatly interested in listening to Dr. Mackintosh's paper, which bristled with points for discussion. Mr. Holland had traversed the ground pretty effectually from the secretary's point of view, but there were several matters he should like to refer to. On the question of the unit of comparison, they all wanted to find a just one, and he sometimes thought they did not attach enough importance to structure. The difference between a hospital such as Charing Cross on a compact triangular site with lifts throughout, and the London spread over 9 acres of ground in the form of a capital E; or, again, between his own hospital at Hampstead and the branch one at Northwood, must be very great in the matter of labour, porters, nursing, and in other ways. He contended that all the different circumstances should be taken into account in instituting comparisons. As to the system of records elaborated by the lecturer, it was the same as they had in London, and he thought they had nothing to learn from the figures he had put before them. No tabulating would alter the difference in the price of coal for instance.

Dr. Mackintosh then replied to the discussion. He requested the last speaker, who "had nothing to learn," to remain, for he wished to convince him of his error. As to the question of accounts, he emphasised the point that under the system he recommended the figures were rendered daily, and he also reminded his hearers that he was able to show the expenditure of each surgeon in detail, as well as how much was spent on each item. He laid great stress on the value of this. He thought it a little unfortunate that Mr. Holland should have said that a man became a medical superintendent simply because he had been a failure as a practitioner. In his own case he had, like Sir Cooper Perry, continued to practise his profession, though he had taken up hospital administration from the first. And, with regard to his scoffing reference to a salary of three or four hundred a year, he might mention that he was getting double that because his efforts were appreciated. Then he had nothing to say against secretaries; they were the manager's best friends; but if they did their own work—getting funds, attending meetings, keeping minutes, and so on—they had no time to do his also.

Mr. Holland remarked that at the London Hospital the secretary had nothing to do with the getting of the money; that was done in another way.

After some further conversation on points of administrative detail, Dr. Mackintosh thanked his audience for the patient hearing accorded him; and the Chairman having expressed to him the indebtedness of those present for his instructive paper, a vote of thanks to the Council of Charing Cross Hospital for the use of the hall brought the proceedings to a close.

